# C-KIT Mutant Anorectal Melanoma: Diagnostic Pitfalls and Treatment Perspectives

**DOI:** 10.7759/cureus.90472

**Published:** 2025-08-19

**Authors:** Hanan Bailal, Soufia El Ouardani, Fadoua Jebrouni, Hind Chibani, Ouissam Al Jarroudi, Sami Aziz Brahmi, Said Afqir

**Affiliations:** 1 Medical Oncology, Mohammed VI University Hospital, Oujda, MAR; 2 Medical Oncology, Faculty of Medicine and Pharmacy, Mohamed First University, Oujda, MAR

**Keywords:** anorectal melanoma, c-kit mutation, metastatic, precision oncology, targeted therapy

## Abstract

Mucosal melanomas (MM) are an uncommon and aggressive subtype of melanoma that differ from cutaneous forms in both genetic profile and clinical behavior. Anorectal melanoma (ARM), in particular, is characterized by its rapid progression and distinct molecular alterations, notably mutations in the *c-KIT* gene. These mutations may serve as potential therapeutic targets and open the door to individualized treatment approaches, especially in the metastatic setting where standard therapeutic options are limited. We report the case of a 72-year-old female admitted to our hospital with a rectal mass noticed during defecation and associated rectal hemorrhage. Vital signs on admission were within normal limits. Histological and molecular biology investigations confirmed ARM harboring a *c-KIT *mutation. The patient received systemic imatinib therapy targeting this molecular alteration, which led to stable disease on imaging and clinical improvement after three months of follow-up. This case study highlights the clinical significance of *c-KIT* mutations in ARM, emphasizing their diagnostic, prognostic, and therapeutic implications.

## Introduction

Mucosal melanomas (MM) are a rare and distinct form of melanoma, accounting for around 1.3% of all cases [1]. They are characterized by their location on mucosal surfaces (anorectal, naso-sinusal, vulva, and vaginal), their aggressive evolution, and their molecular profile, which differs from cutaneous forms [2]. Anorectal melanoma (ARM) is an aggressive MM, accounting for 0.4-1.6% of all melanomas [3]. This entity is distinguished from classic cutaneous melanomas by a specific molecular profile, notably the *c-KIT* gene mutation observed in 35.5% of cases [4]. ARM with *c-KIT* mutation opens the way to targeted therapeutic options, although no consensus has yet been reached on the optimal strategy [5]. We present a case of ARM harboring an activating *KIT* mutation, with the aim of exploring the clinical, pathological, and molecular features of this rare malignancy while highlighting the prognostic and therapeutic implications of *c-KIT* mutations.

## Case presentation

In November 2024, a 72-year-old female with a medical history of two ischemic strokes on anticoagulation therapy, insulin-dependent diabetes mellitus, and hypertension under medical treatment was referred to the Regional Oncology Center of Oujda, Morocco, for management of an ARM. The patient reported noticing a rectal mass during defecation, accompanied by intermittent rectal hemorrhage. Upon admission, her vital signs were within normal limits, indicating a stable general condition. Pelvic MRI revealed two tumor lesions in the lower rectum. The first lesion, measured 30 × 26 × 24 mm, was located on the left posterolateral wall of the lower rectum and penetrated the left internal sphincter while preserving the external sphincter, particularly the left levator ani muscle, and the intersphincteric fat space. The second lesion, measured 15 × 11 × 9 mm, was located in the left lateral rectal wall and had no extra-parietal extension; its lower pole was 49 mm from the anal verge.

**Figure 1:**
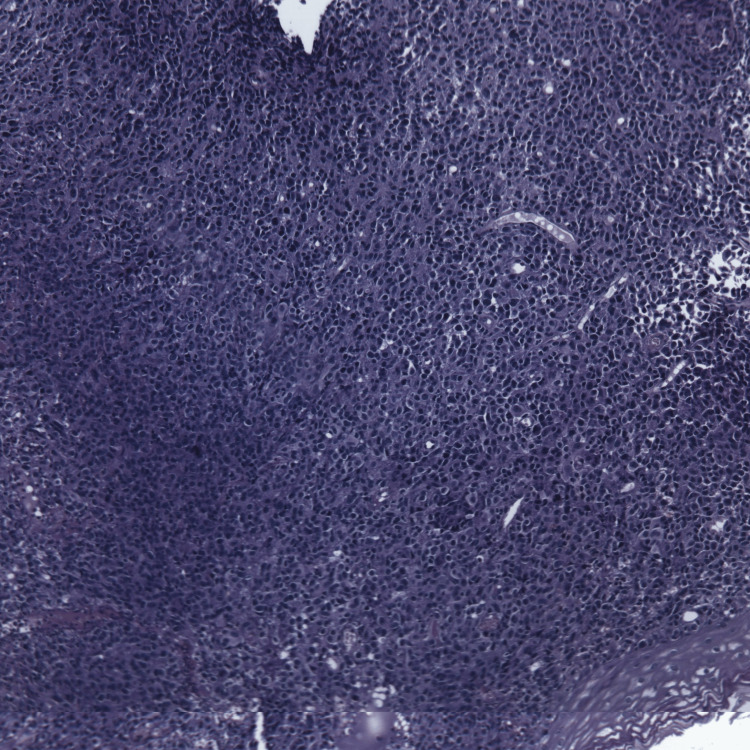
Tumor cells with abundant cytoplasm and round or oval hyperchromatic nuclei GAB x10

Histopathological examination of biopsy specimens revealed tumor cells with pale cytoplasm, round to oval hyperchromatic nuclei, and occasional prominent nucleoli (Figure 1).

**Figure 2:**
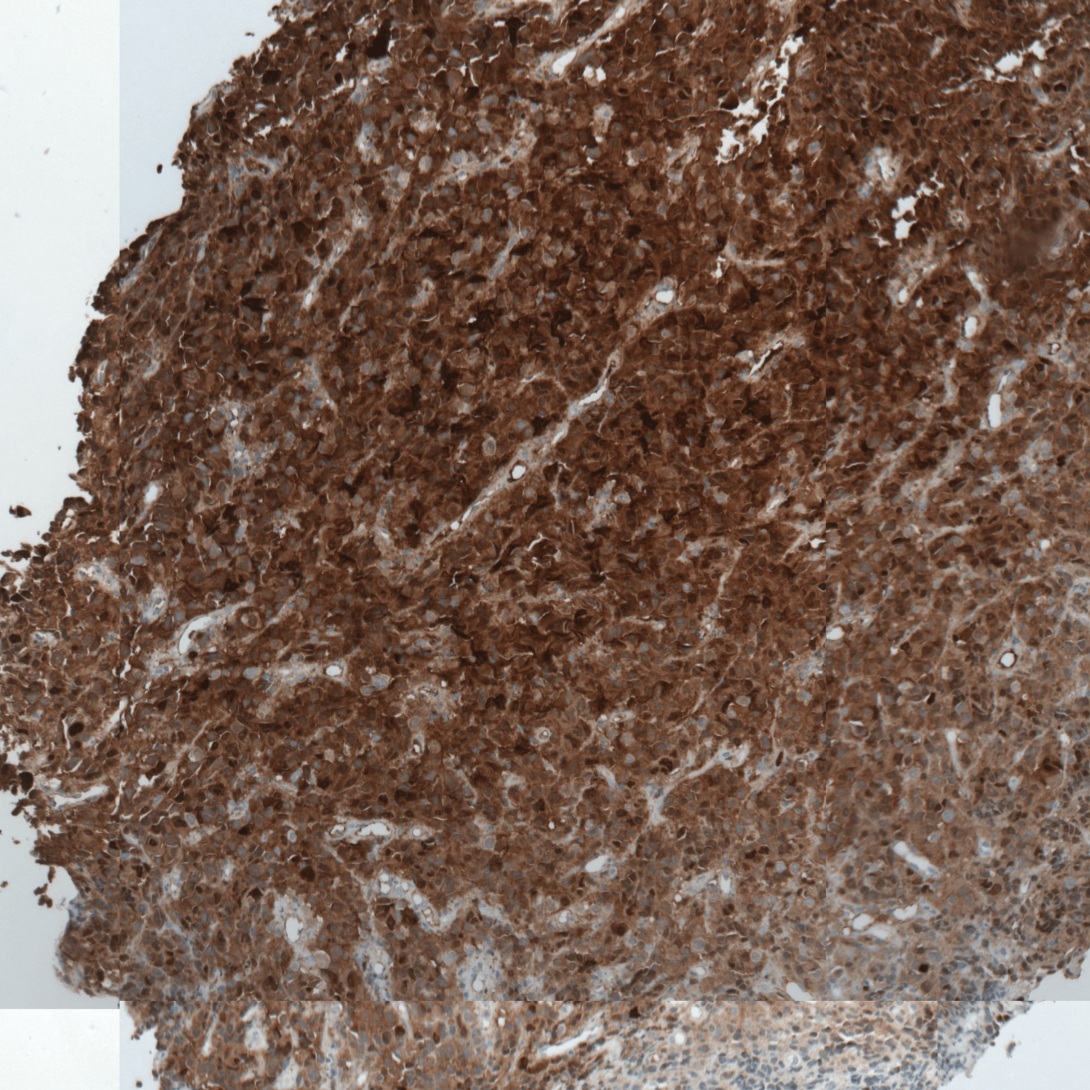
Positive labeling of tumor cells by PS100 GAB x10

**Figure 3:**
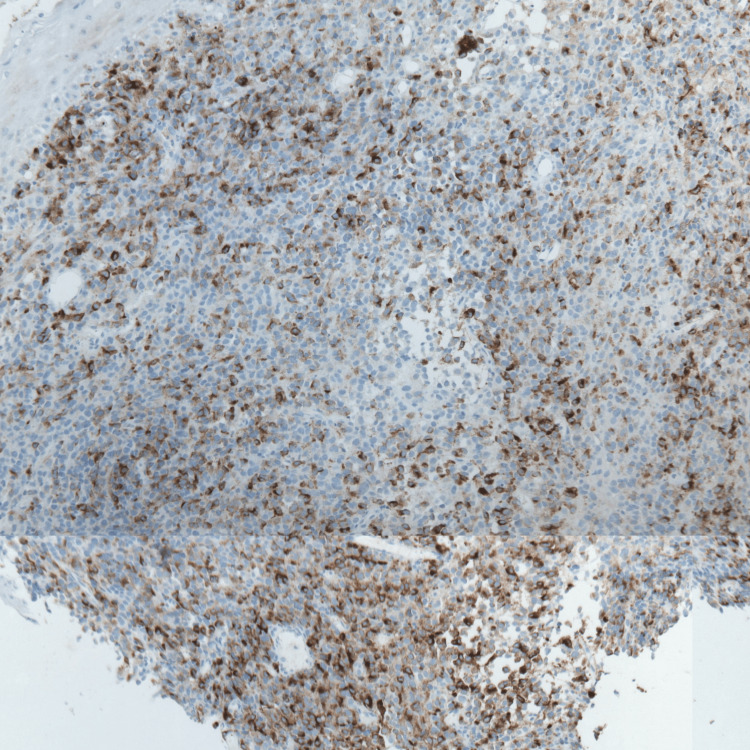
Positive labeling of tumor cells by HMB45 GAB x10

**Figure 4:**
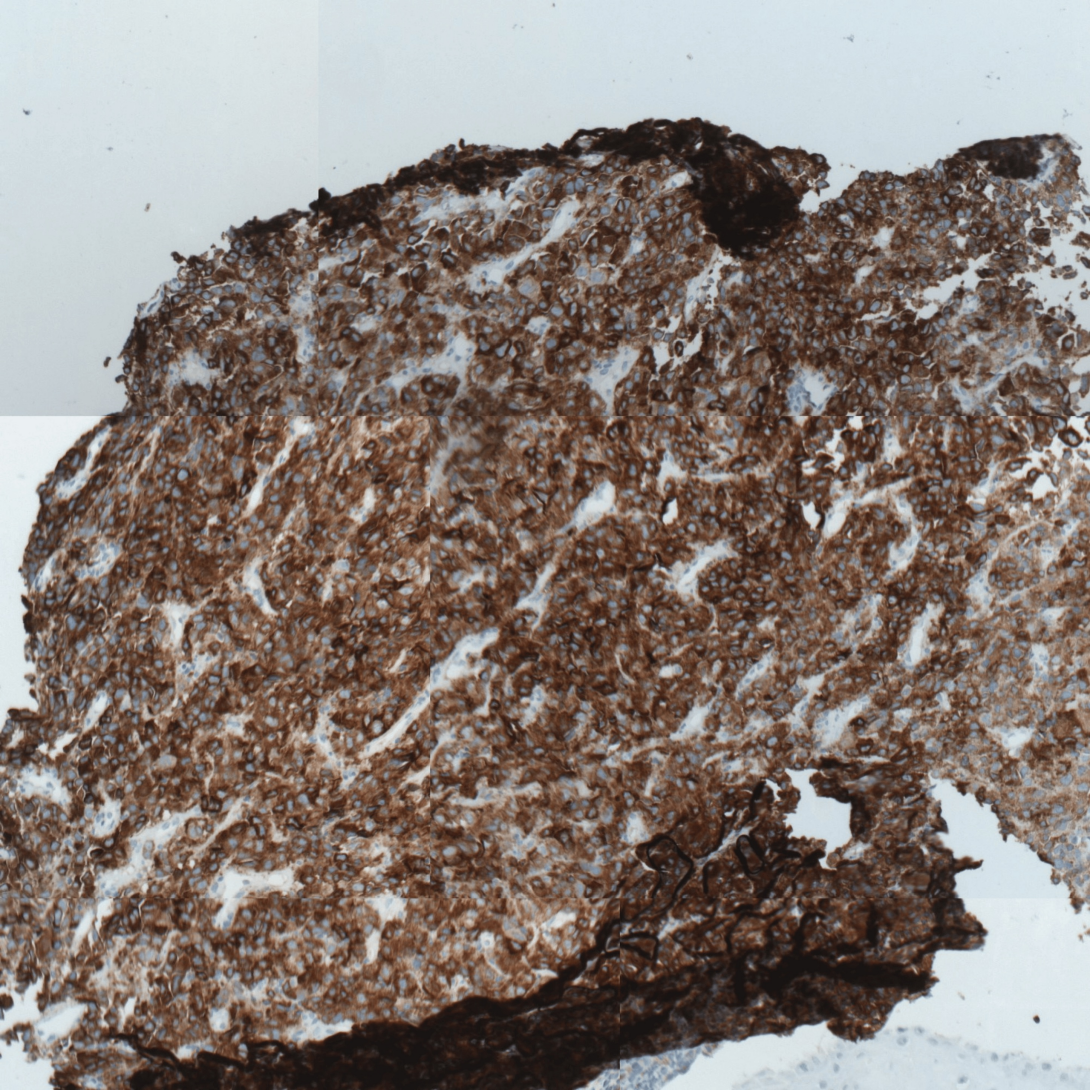
Positive labeling of tumor cells by Melan A GAB x10

Immunohistochemistry demonstrated strong positivity for S-100, HMB-45, and Melan A (Figures 2-4), confirming the diagnosis of malignant melanoma. Next-generation sequencing (NGS) identified an activating mutation in exon 11 of the *KIT *gene.

Based on this molecular finding, the patient was started on imatinib 400 mg daily, with monthly follow-up. Treatment was well tolerated clinically and biologically, and radiological assessments demonstrated stable disease after three months of therapy.

## Discussion

MM are uncommon tumors compared to cutaneous melanomas [1]. Based on cancer statistics for the United States in 2023, melanomas account for 5% of all cancers, of which ≤2% are MM [6]. Unlike cutaneous melanomas, whose etiology is often linked to ultraviolet (UV) exposure, MM arise on sun-protected surfaces, suggesting distinct tumorigenic mechanisms [7]. MM arise from melanocytes located within mucous membranes of the body and are distributed, in decreasing order of frequency, as follows: approximately 50% occur in the head and neck region, 37% in the gastrointestinal tract, 6% in the female genital tract, and 4% in the urinary tract [8]. ARM represents approximately 0.4-1.6 %of all melanomas, accounts for 238 % of MMs, and comprises about 1% of all colorectal cancers [3]. ARM is most commonly diagnosed in individuals over 50 years old, with a higher incidence in females than in males [9].

ARM typically presents with vague and insidious symptoms, often leading to a delayed diagnosis and an unfavorable prognosis. Unlike cutaneous melanomas, its clinical manifestations are commonly misinterpreted as benign anorectal disorders, such as hemorrhoids or anal fissures [3]. The most common symptoms are rectal bleeding, anal pain, a palpable mass, altered bowel functions, and, rarely, tenesmus [5]. In some cases, the lesion is incidentally discovered during a routine rectal examination or endoscopy. Because of its submucosal growth pattern and often amelanotic appearance, the tumor may go undetected both clinically and histologically [10]. Diagnosis is typically established through colonoscopy and biopsy. Typically, it presents as a pigmented lesion, which may appear as a macule, papule, or exophytic nodular lesion, with irregular borders and heterogeneous coloration, occasionally amelanotic with a reddish appearance [9].

The definitive diagnosis is established through histopathological examination combined with immunohistochemistry [9]. The pathological analysis may reveal the growth of spindle-shaped or epithelioid cells with a high rate of mitosis and noticeable nuclear atypia [11]. Classically, the immunohistochemistry reveals strong positivity for melanocytic markers, such as human melanoma black 45 (HMB-45), Melan A, S100 protein, and SOX10 (SRY (Y sex determination region) - Box 10) [11]. ARM are clearly distinguished from cutaneous melanomas by a lower frequency of *BRAF *mutations and a higher prevalence of *c-KIT *gene alterations, observed in around 35.5% of cases [4]. The *c-KIT *mutation, which affects a receptor tyrosine kinase, leads to constitutive activation of the *c-KIT *tyrosine kinase receptor, independently of its physiological ligand, the stem cell factor (SCF). This continuous activation stimulates key intracellular signaling pathways, notably the MAPK/ERK and PI3K/AKT pathways involved in cell proliferation and survival, and is a biomarker of both prognostic and therapeutic interest [12].

Management strategies of metastatic ARM are largely extrapolated from data on metastatic mucosal and cutaneous melanomas, due to the rarity of the disease and the lack of dedicated clinical trials [3]. Systemic therapy remains the preferred treatment in the metastatic setting. Patients with metastatic rectal melanoma harboring an activating *c-KIT *mutation may benefit from a targeted approach based on the use of tyrosine kinase inhibitors (TKIs) [13]. Among these agents, imatinib has demonstrated significant antitumor activity, particularly in patients with mutations in exons 11 or 13 of the *KIT *gene. This targeted therapy can lead to durable clinical responses in some cases, although its efficacy varies depending on the type of mutation [13,14]. In parallel, immunotherapy with immune checkpoint inhibitors (anti-PD-1 or anti-PD-1/anti-CTLA-4 combination) may be considered, particularly in cases of progression under TKI or in forms without a targetable mutation. Initial molecular profiling is therefore essential to guide therapeutic choice, and management must be individualized, ideally within a multidisciplinary approach [4,13].

## Conclusions

Metastatic rectal melanoma remains a rare and particularly aggressive form of melanoma, with limited therapeutic options. This case highlights the value of thorough molecular analysis - especially the identification of activating *c-KIT* mutations - which can open the door to targeted therapies, such as TKIs. In selected cases, these treatments may offer encouraging clinical responses. Through this case, we emphasize the importance of an individualized approach, based on both the clinical and molecular characteristics of the tumor, in order to optimize patient management. Sharing such clinical observations contributes to the growing understanding of this still poorly understood disease.
